# Therapeutic Effect of *Lactiplantibacillus plantarun* HFY11 Isolated from Naturally Fermented Yak Yogurt on Lincomycin Hydrochloride–Induced Diarrhea in Mice

**DOI:** 10.3390/microorganisms12112307

**Published:** 2024-11-13

**Authors:** Fang Tan, Lixuan Ren, Chang-Suk Kong

**Affiliations:** 1 Department of Bioscience, Silla University, Busan 46958, Republic of Korea; g-202311663@sillain.ac.kr (F.T.); g-202311664@sillain.ac.kr (L.R.); 2 Department of Food Science and Nutrition, Silla University, Busan 46958, Republic of Korea; 3 Marine Biotechnology Center for Pharmaceuticals and Foods, Silla University, Busan 46958, Republic of Korea

**Keywords:** *Lactiplantibacillus plantarun*, diarrhea, lincomycin hydrochloride, mice, expression

## Abstract

This study aimed to observe the therapeutic effect of *Lactiplantibacillus plantarun* HFY11 (LP-HFY11) on lincomycin hydrochloride-induced diarrhea in mice. The results showed that LP-HFY11 alleviated weight loss and intestinal and colon tissue lesions caused by diarrhea. The serum assay showed that LP-HFY11 decreased interleukin 17A (IL-17A), IL-6, 5-hydroxytryptamine, and malondialdehyde levels and increased total antioxidant capacity in mice with diarrhea. LP-HFY11 also downregulated the mRNA expression of cystic fibrosis transmembrane conductance regulator (CFTR), epidermal growth factor receptor (EGFR), and transforming growth factor beta 1 (TGFβ1) and upregulated the expression of recombinant sodium/hydrogen exchanger 1 (NHE1) and NHE4 in the colon tissues of mice with diarrhea. In conclusion, the study showed that LP-HFY11 could effectively inhibit diarrhea, and the effect was better than that of the drug *Bifidobacterium* tetragenous viable bacteria tablets (*Bifidobacterium*-TVBT).

## 1. Introduction

A strong relationship exists between diarrhea and intestinal health. Diarrhea is a digestive disorder characterized by loose or liquid stools and an increased frequency of bowel movements. It is often caused by intestinal infections, food poisoning, allergic reactions, side effects of drugs, or inflammation of the bowel. A certain association has been reported between diarrhea and antibiotics [1]. Antibiotics are drugs used to treat bacterial infections. They not only kill the bacteria that cause infection but also destroy the beneficial flora in the gut. This imbalance of flora leads to intestinal dysfunction, causing dyspepsia and diarrhea. Diarrhea is often accompanied by substantial fluid loss, especially when it occurs for a prolonged duration [2]. This leads to dehydration and loss of water and electrolyte balance in the body. Diarrhea may lead to inflammation of the bowel, such as inflammatory bowel disease. These inflammatory diseases cause chronic diarrhea, abdominal pain, and other kinds of discomfort; in severe cases, they even affect bowel function and overall health [3].

Plateau yak yogurt is a unique fermented dairy product, which is mainly made from plateau yak milk. Compared with ordinary yoghourt, plateau yak yoghourt has some unique advantages in microbial composition [4]. Plateau yaks are native to the plateau region, and this special growth environment contributes to the unique tolerance of microorganisms in their dairy products. These microorganisms can still ferment well under low oxygen and low temperature conditions and have stronger adaptability [5]. Microbial metabolites (such as lactic acid and alcohols) in plateau yak yogurt can inhibit the growth of harmful microorganisms and have certain natural antiseptic properties [6]. The probiotics and fermentation products in plateau yak yogurt help to enhance immunity, improve digestive function, reduce cholesterol, etc., and have a positive effect on overall health [7,8,9].

Lactic acid bacteria are a kind of probiotic bacteria. Lactic acid bacteria are widely distributed, usually found in meat, milk and vegetables and other foods and products. *Lactiplantibacillus plantarun* is a kind of lactic acid bacteria, which has a positive impact on the living environment of intestinal microorganisms. It can colonize the intestine and play a beneficial role. For example, it can produce a large amount of acid, thus inhibiting the colonization and propagation of intestinal harmful bacteria, promoting the propagation of beneficial intestinal bacteria, regulating the balance of intestinal flora and maintaining the stability of intestinal flora. It is also helpful to improve intestinal flora disorders caused by constipation, diarrhea and other uncomfortable symptoms [1,4,5]. The Qinghai–Tibet Plateau of China has a unique natural environment, and local herdsmen have been eating naturally fermented yak yogurt for more than 1000 years. *Lactiplantibacillus plantarun* HFY11 (LP-HFY11), a lactic acid bacteria isolated from naturally fermented yak yogurt on the Qinghai–Tibet Plateau at an altitude of more than 4000 m, has strong in vitro ability to resist gastric acid and bile salt, and is expected to colonize the intestinal tract well. Thus, it exerts its biological activity.

The intestinal tract has a barrier function and is also the main place for the digestion and absorption of nutrients in the body. The normal intestinal barrier function includes the intestinal flora and endocrine system, the intestinal immune system, and the intestinal mucosal epithelial barrier; the most important of these three functions is the intestinal mucosal epithelial barrier [10]. If the intestinal mucosa is damaged, the water and salt balance is disrupted, the permeability increases, and the intestinal flora is disordered, which can cause intestinal inflammatory diseases, reduce physiological activities, and even lead to death [11]. Probiotics provide beneficial flora to restore the normal composition of intestinal flora, thereby enhancing the intestinal barrier function, protecting the intestinal mucosa, preventing the invasion of harmful substances and pathogenic bacteria, and alleviating the symptoms of diarrhea [12]. *Bifidobacterium* tetragenous viable bacteria tablet is a clinical drug used to treat diarrhea associated with intestinal dysbiosis. This study used it as a positive control drug for investigating diarrhea due to intestinal dysbiosis caused by antibiotics. To our knowledge, this was the first in vivo study of LP-HFY11, a newly discovered strain of naturally fermented yak yogurt from Qinghai–Tibet Plateau, China, to investigate its role in inhibiting diarrhea and verify its potential application as an anti-diarrheal probiotic.

## 2. Materials and Methods

### 2.1. Isolation and Purification of Lactic Acid Bacteria

The yogurt samples in this study were traditional naturally fermented yak yogurt collected by the team from the homes of herdsmen in the Hongyuan County area of Aba Tibetan and Qiang Autonomous Prefecture, Sichuan Province, China. A 1 mL yogurt sample was taken and diluted in sterile physiological saline solution to a 10-fold gradient (10^−6^). The plates were then incubated at 37 °C for 24–48 h, and the colony morphology was recorded and observed. Colonies of different morphologies were selected and then streaked onto fresh plates.

### 2.2. Preliminary Identification of Lactic Acid Bacteria

The supernatant was disposed of after the bacterial pellet was resuspended in a sterile physiological saline solution. When Gram staining was first performed, bacteria showing a positive result were first believed to be lactic acid bacteria.

### 2.3. Polymerase Chain Reaction Amplification of Genomic DNA and Agarose Gel Electrophoresis Detection

DNA was extracted using a bacterial genomic DNA extraction kit (Solarbio Life Sciences, Beijing, China). The obtained DNA was amplified through polymerase chain reaction (PCR) using specific primers. Further, 1 µL each of DNA was added for the downstream primer 1495R (5′-CTACGGCTACCTTGTTACGA-3′) and the upstream primer 27F (5′-AGAGTTTGATCCTGGCTCAG-3′). The amplified PCR products were sent to Beijing Qengke Biotechnology Co., Ltd. (Beijing, China) for sequencing. The obtained sequences were aligned and assessed using the Basic Local Alignment Search program available in the NCBI database. The identified strains were deposited in China General Microbiological Culture Collection Center (Beijing, China), the preservation number is CGMCC No.16644.

### 2.4. Tolerance of Lactic Acid Bacteria to 0.3% Bile Salt

Pig bile salt was added to MRS-THIO medium (Solarbio Life Sciences) to achieve a concentration of 0.3%. The OD_600 nm_ values of the various medium concentrations were measured after a 24 h incubation at 37 °C. The following formula was used to calculate the bile salt tolerance of the strain: tolerance (%) = (OD_600 nm_ of 0.3% bile salt media − OD_600 nm_ of control)/(OD_600 nm_ of 0.3% bile salt medium − OD_600 nm_ of control) × 1000.

### 2.5. Artificial Gastric Juice Tolerance Test

A solution of 0.2% sodium chloride and 0.35% pepsin was used to obtain the artificial gastric juice. After serially diluting the samples 10 times in 0 and 3 h, the live bacteria were counted using the pour plate method. The plates were incubated in a solid MRS medium at 37 °C for 48 h. The survival rate (%) was calculated using the following formula: survival rate (%) = (number of viable bacteria in 3 h number of viable bacteria in 0 h) × 100.

### 2.6. Animal Experiment

A total of 50 male SPF-grade BALB/c mice, weighing 25–30 g and aged 7 weeks, were obtained from Chongqing Medical University Animal Experiment Center (Chongqing, China), with animal license number SCXK (Yu) 2022-0010. Ten BALB/c mice each were randomly assigned to the following five groups: model, normal, LP-HFY11 high-concentration therapy (LP-HFY11H), *Bifidobacterium* tetragenous viable bacteria tablets (*Bifidobacterium*-TVBT, positive control group), and LP-HFY11 low-concentration treatment (LP-HFY11L). From the first day of the experiment, the mice in all groups except the normal group were given 120 mg/kg lincomycin hydrochloride oral via gavage every day for seven consecutive days. The mice in the normal group were given the same dose of normal saline. At the same time, the mice in the *Bifidobacterium*-TVBT group were fed 0.1% (*w*/*w*) *Bifidobacterium* hydrochloride tetragenous viable bacteria tablet powder and drinking water freely. The mice in the LP-HFY11L and LP-HFY11H groups were fed 0.01% (*w*/*w*, 10^7^ CFU LP-HFY11/g feed) and 0.1% (*w*/*w*, 10^8^ CFU LP-HFY11/g feed) LP-HFY11 lyophilized powder and water ad libitum for 7 days, respectively. The body weight of mice was measured and recorded daily. On day 8, all mice were fasted for 12 h and sacrificed. The serum, small intestine, and colon were collected after dissection. Every operation followed the guidelines for breeding experimental mice, and other experimental techniques complied with the ethical standards for using experimental animals (Collaborative Innovation Center for Child Nutrition and Health Development approval number: 202402021B).

### 2.7. Hematoxylin and Eosin Staining of Sections of Intestinal Tissues

The intestinal tissues (small intestine and colon) of mice were fixed by immersion in neutral formalin, embedded, sectioned, stained, and prepared. Finally, the pathological changes were observed under a light microscope (BX53, Olympus, Tokyo, Japan), and the images were taken [13].

### 2.8. Determination of Serum Cytokine and Antioxidant Levels in Mice

The whole blood of mice was centrifuged at 14,000 rpm for 10 min at 4 °C in a high-speed freezer centrifuge, and the supernatant of mouse serum was collected to repeat the procedure three times. The supernatant after the third centrifugation was used to determine the total antioxidant capacity (T-AOC) and the levels of malondialdehyde (MDA), 5-hydroxytryptamine (5-HT), mouse interleukins (IL-6 and IL-17) in the serum of mice following the instructions on the corresponding kits (Thermo Fisher Scientific, Waltham, MA, USA).

### 2.9. Determination of Gene Expression Using Reverse Transcription–PCR

After crushing mouse tissue, RNAzol reagent (Solarbio Life Sciences) was used to separate the whole RNA from the colon and small intestine tissue. The total RNA concentration was reduced to 1 μg/μL. Then, 1 μL of oligodT18, RNase, deoxy-ribonucleoside triphosphate (dNTP), murine leukemia virus (MLV) enzyme, and 10 μL of 5× buffer (Thermo Fisher Scientific) were mixed with 2 μL of diluted total RNA extract. The cDNA was produced under the following conditions: 37 °C for 120 min, 99 °C for 4 min, and 4 °C for 3 min. The reverse transcription (RT)-PCR was then used to increase the mRNA expression (Table 1, Thermo Fisher Scientific). Meanwhile, the housekeeping gene GAPDH was amplified under the same conditions and used as an internal reference. Ultimately, the 2^−ΔΔCt^ method was employed to determine the relative expression level of every gene [14,15].

### 2.10. Statistical Analysis

The average value of the parallel experiments was determined following three repetitions. The data from each group were analyzed for significant differences using SAS 9.1 statistical software by one-way analysis of variance at a *p* value less than 0.05.

## 3. Results

### 3.1. Determination of Lactic Acid Bacteria in the Sample

**Figure 1:**
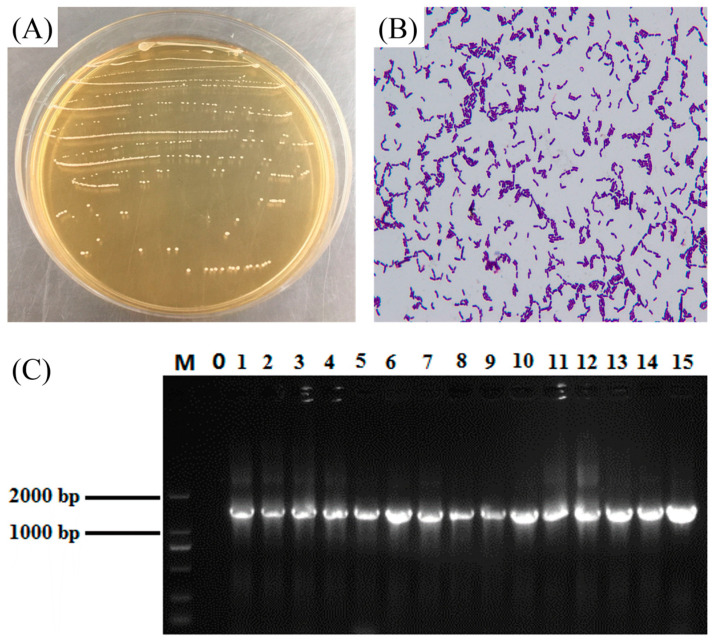
Colony morphology (**A**), gram staining morphology (**B**) and 16S rDNA agarose gel electrophoresis of PCR amplification products (**C**) of *Lactiplantibacillus plantarun* HFY11. M: D2000 DNA Marker; 0: Negative control group; 11: *Lactiplantibacillus plantarun* HFY11.

Figure 1A depicts the microbial colonies isolated and purified from the yak yogurt sample, typically white or off-white with a round shape, clean edges, and a wet, smooth surface. The cells were rod-shaped, and no budding reproduction was observed. Gram staining showed a positive result (Figure 1B). Figure 1C shows the 1.2% agarose gel electrophoresis findings for the 16S rDNA amplification. The lane containing sterile ultrapure water as a negative control revealed no bands, demonstrating no contamination during PCR amplification. The amplification of fragment length in the lane was around 1500 bp, which was in line with the predicted amplification fragment length. The sequencing results showed 100% homology with the known strains of *Lactiplantibacillus plantarun* in the GeneBank database. Thus, the isolated strain from the sample was recognized as *L. plantarum* and classified as HFY11 based on morphological observations and 16S rDNA species analysis. LP-HFY11, isolated by our research group from naturally fermented yak milk in the homes of herders in Hongyuan County, Aba Tibetan-Qiang Autonomous Prefecture, Sichuan Province, China, is now housed at the China General Microbiological Culture Collection Center (CGMCC) under the accession number CGMCC No. 16644.

### 3.2. In Vitro Resistance of L. plantarum HFY11

LP-HFY11 exhibited 82.15% viability in pH 3.0 artificial gastric juice and 51.35% growth efficiency in 0.3% bile salt (Table 2). This indicated that the strain could tolerate artificial gastric acid and bile salts, demonstrating good in vitro resistance. These findings provided initial conditions for further development of LP-HFY11 as a probiotic. Consequently, animal experiments were conducted to validate the intervention effect of LP-HFY11 on diarrhea.

### 3.3. Body Weight Change in Mice

**Figure 2:**
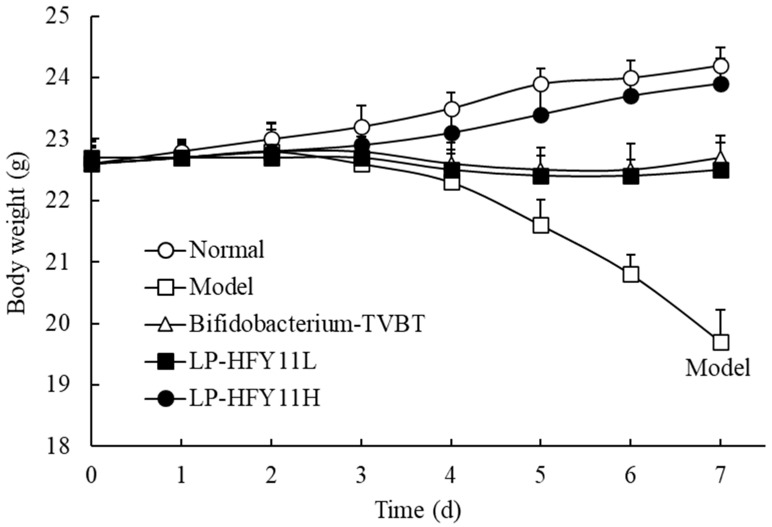
Weight changes in mice during the experiment.

As shown in Figure 2, the weight of mice gradually decreased over time in the diarrhea group compared with the normal group. After feeding LP-HFY11 bacteria powder, the weight of mice with diarrhea increased significantly, but it was still lower than that in the normal group. The *Bifidobacterium*-TVBT group showed the same trend as that in the low-dose LP-HFY11 group. Moreover, the effect of high-dose LP-HFY11 was significantly better than that in the *Bifidobacterium*-TVBT group.

### 3.4. Serum Index Detection in Mice

**Figure 3:**
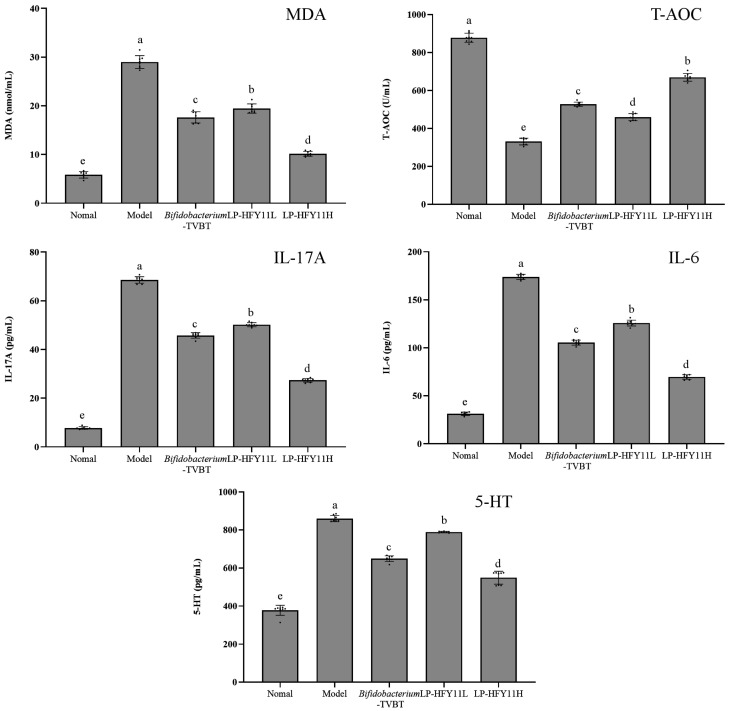
Changes in IL-17A, IL-6, 5-HT, T-AOC, and MDA in serum of mice. ^a–e^ The letters denote statistically significant differences (*p* < 0.05) in the data mean values between the groups.

Figure 3 shows the changes in T-AOC and the levels of IL-17A, IL-6, 5-HT, and MDA in the serum of mice. The expression levels of pro-inflammatory factors IL-6, IL-17A, MDA, and gastrointestinal hormones were high in the serum of mice in the diarrhea model group. The levels significantly decreased after intervention with LP-HFY11 and *Bifidobacterium*-TVBT (*p* < 0.05). The T-AOC in the serum of mice was significantly increased in the LP-HFY11H group compared with the diarrhea group, and was higher than that in the *Bifidobacterium*-TVBT and LP-HFY11L groups (*p* < 0.05).

### 3.5. Pathological Observation of the Small Intestine of Mice

**Figure 4:**
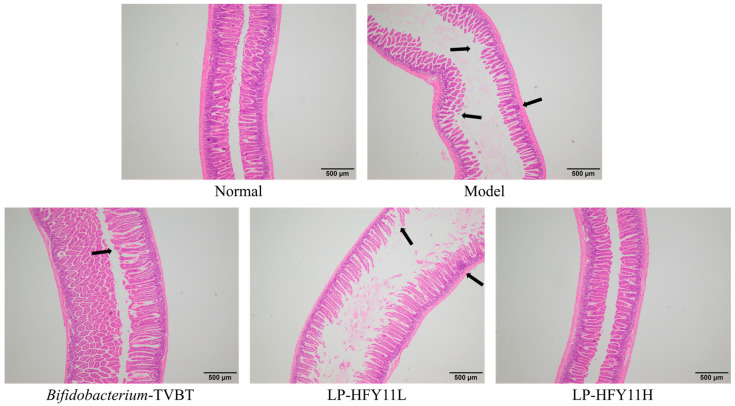
Observation of mouse small intestine Sections (4×). The location indicated by the arrow is the site of tissue lesion.

As shown in Figure 4, the intestinal villi were intact, and the intestinal epithelial cells were closely arranged in the normal group. In the model group, the villi and epithelial structures of the small intestine were destroyed, accompanied by edema and necrosis. The intestinal villi of mice in the *Bifidobacterium*-TVBT group were found to be intact under the microscope; the villi were wider than those in the normal group. Some of the villi were broken or dissolved. The villi of the small intestine in the LP-HFY11H group were widened and relatively intact. The intestinal structure in the LP-HFY11L group was similar to that in the *Bifidobacterium*-TVBT group.

### 3.6. Pathological Observation of the Colon of Mice

**Figure 5:**
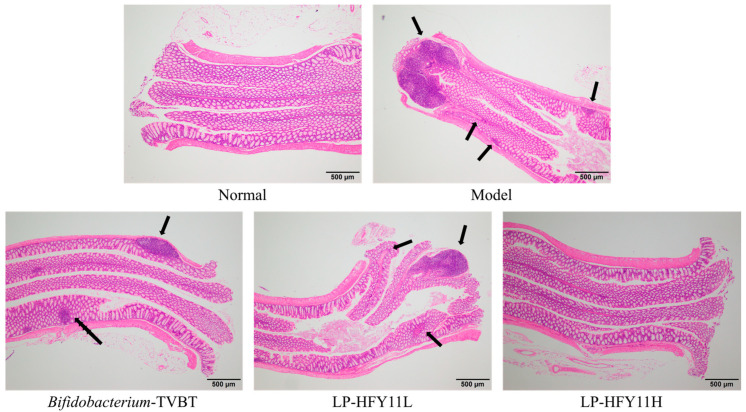
Observation of mouse colon Sections (4×). The location indicated by the arrow is the site of tissue lesion.

The structure of the colon was complete, the epithelial cells were arranged neatly, and the tree-like structure was clear in the normal group (Figure 5). In the model group, the colon tissue structure was extremely damaged, and the intestinal villi were severely shed or broken, accompanied by pathological changes or necrosis. The intestinal villi were significantly wider, the structure was intact, no obvious edema was observed, and no obvious shedding or fusing occurred in the *Bifidobacterium*-TVBT group compared with the normal group, except for a small amount of inflammatory cell infiltration. The intestinal villi in the LP-HFY11H group were wider than those in the normal group, and the intestinal epithelial cells were relatively intact.

### 3.7. mRNA Expression of Related Genes in the Small Intestine and Colon of Mice

**Figure 6:**
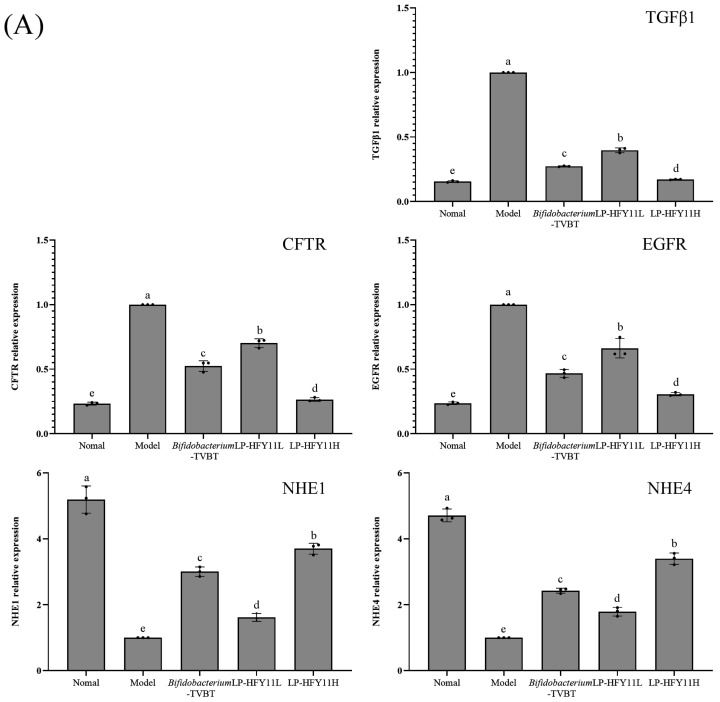

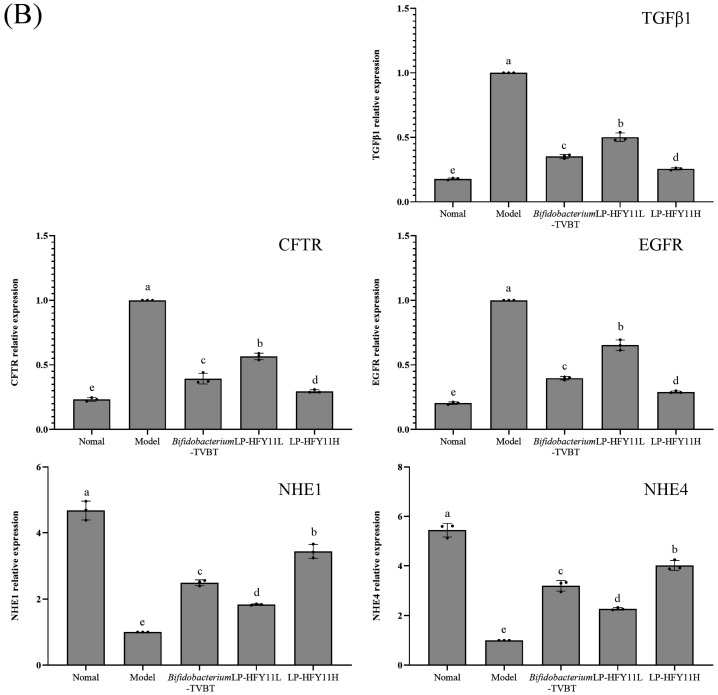
Changes in CFTR, EGFR, TGFβ1, NHE1, and NHE4 in mRNA expression of mice small intestine (**A**) and colon (**B**) tissues. ^a–e^ The letters denote statistically significant differences (*p* < 0.05) in the data mean values between the groups.

Figure 6A,B shows that the expression levels of cystic fibrosis transmembrane conductance regulator (CFTR), epidermal growth factor receptor (EGFR), and transforming growth factor beta 1 (TGFβ1) were high in the small intestine and colon tissues of mice in the model group, whereas the expression levels of sodium/hydrogen exchanger 1 (NHE1) and NHE4 significantly decreased compared with those in the normal group. The expression levels of CFTR, EGFR, and TGFβ1 in the tissues significantly decreased, while the expression levels of NHE1 and NHE4 significantly increased, in the LP-HFY11H, *Bifidobacterium*-TVBT, and LP-HFY11L groups, compared with the model group (*p* < 0.05). The expression levels in the LP-HFY11H and normal groups were most similar.

## 4. Discussion

The resistance of bacterial strains in pH 3.0 artificial gastric acid and 0.3% bile salt is a basic in vitro evaluation method for assessing the probiotic potential of bacterial strains [14]. LP-HFY11 has good anti artificial gastric acid and bile salt ability, and preliminary evaluation suggests that it has probiotic potential. Therefore, this study continues to use animal models to evaluate its application in intervening in diarrhea.

The loss or destruction of intestinal mucosa can reduce the body’s ability to digest and absorb, and the ability to fight against the invasion of antigens in vitro, leading to many intestinal diseases [16]. The diarrhea model was induced by lincomycin hydrochloride injection in this study. Lincomycin can cause dysbiosis of the gut microbiota, lincomycin can kill bacteria that are beneficial to human health in the intestine, causing bacterial flora disorders, food metabolism disorders, and osmotic diarrhea. The imbalance of flora destroys the protective barrier of the intestinal mucosa, leading to infectious diarrhea [17]. After diarrhea, the body suffers from dehydration, loss of appetite, dyspepsia, and other diseases, resulting in weight loss. In this experiment, the weight of mice with diarrhea also decreased significantly. LP-HFY11 inhibited the weight loss caused by diarrhea and alleviated the disease.

Antibiotic-induced diarrhea is often accompanied by systemic inflammation and an increase in the secretion of pro-inflammatory cytokines IL-6 and IL-17A [18]. T-AOC is one of the indexes to measure the level of oxidative stress in the body. During diarrhea, the oxygen free radicals in the body increase dramatically, triggering a chain reaction of free radicals, causing lipid peroxidation and an increase in MDA levels [19]. 5-HT is one of the essential neurotransmitters present in the gastrointestinal tract. Its expression increases when diarrhea occurs in the body, which also causes contraction in the gastrointestinal tract [20]. The experimental results showed that LP-HFY11H effectively adjusted the T-AOC and the levels of IL-6, IL-17A, MDA, and 5-HT in mice with diarrhea, thus maintaining intestinal health and alleviating symptoms of diarrhea.

Diarrhea can cause soft tissue damage, which may stimulate the intestinal mucosa and lead to rupture and bleeding of the intestinal mucosa, causing intestinal ischemia. In severe cases, it can also induce bacterial infection and cause enteritis, resulting in pathological damage to intestinal tissue. Therefore, hematoxylin and eosin staining observations of intestinal tissue pathology is also an important means of checking the degree of diarrhea [21]. This study also confirmed that LP-HFY11 can effectively alleviate intestinal tissue lesions caused by diarrhea, intervene in diarrhea, and promote body recovery.

The disturbance of electrolyte balance in the gastrointestinal tract and increased water content in the stool often induce diarrhea. The electroneutral pathway mediated by Na^+^/H^+^ exchangers (NHEs) located on the side of the intestinal lumen can absorb a large amount of NaCl [22]. NHE1 and NHE4 are the carriers playing an important role in the electroneutral sodium absorption in the intestine [23]. TGF-β1 is a multifunctional cytokine that regulates the growth and differentiation of various cell types, promotes inflammation, and inhibits immunity [24]. CFTR regulates cystic fibrosis transmembrane transduction, which mainly regulates cell water content by activating the chloride pathway, whereas EGFR can also cause intestinal edema by inhibiting cell growth [25]. The experimental data showed that LP-HFY11 regulated mRNA expression in the small intestine and colon so that the intestinal tissue could restore normal function.

Probiotics help maintain the balance of microbes in the gut and inhibit the growth of pathogenic bacteria by competing with harmful bacteria for nutrients and living space. This microecological balance is essential for digestive and absorptive functions [26]. Probiotics can promote the growth and repair of intestinal epithelial cells, enhance the integrity of the intestinal barrier, and reduce harmful substances and inflammatory factors entering the blood through the intestinal barrier. This helps prevent diseases caused by increased intestinal permeability [27]. Probiotics can help restore the balance of microbes in the gut. Diarrhea often leads to the reduction in beneficial bacteria and the increase in pathogenic bacteria in the gut. Probiotics help to restore a healthy microecological environment by using competition, inhibiting the growth of pathogenic bacteria, and enhancing the immune response [28]. Probiotics can stimulate the intestinal immune system and enhance the body’s resistance to infection, thereby reducing the risk of diarrhea, especially acute diarrhea caused by viruses or bacteria [29]. Probiotics can promote the repair and regeneration of intestinal epithelial cells and enhance the integrity of the intestinal barrier, thereby reducing the penetration of harmful substances and pathogens and reducing the possibility of diarrhea [30]. When treated with certain antibiotics, probiotics can reduce the discomfort of antibiotic-associated diarrhea (AAD) and enhance the intestinal recovery capacity [12]. Other studies have shown that probiotics can alleviate the uncomfortable symptoms caused by diarrhea, such as abdominal pain and bloating, and improve the quality of life [31]. LP-HFY11 could reduce inflammation, regulate intestinal tract and inhibit diarrhea, suggesting that LP-HFY11 could be used as a novel probiotic.

## 5. Conclusions

With diarrhea, the gut may have fewer good bacteria and more harmful bacteria. Probiotics can help restore the balance of healthy flora in the gut and inhibit the growth of harmful bacteria [32]. Probiotics can enhance the tight junction of intestinal epithelial cells, improve intestinal barrier function, and reduce intestinal leakage, thereby reducing the risk of infection and inflammation. Probiotics can reduce the occurrence of diarrhea by regulating the response of the intestinal immune system. They enhance the activity of immune cells in the gut and help fight pathogens [33]. Some probiotics, especially lactic acid bacteria, produce short-chain fatty acids, which reduce intestinal discomfort and relieve symptoms caused by diarrhea [34]. In summary, LP-HFY11 is a microbe which plays an essential role in the gut. It enhances intestinal function, restores the intestinal structure to its normal state, protects the intestinal structure, and promotes intestinal motility. In addition, it also regulates the disordered intestinal flora and relieves diarrhea in mice, thus potentially serving as an anti-diarrheal probiotic. This study has preliminarily verified the intervention effect of LP-HFY11 on diarrhea. In the future, it is necessary to strengthen relevant studies in animal and human clinical practice, observe the changes in intestinal flora by sequencing the intestinal microbes, and verify the effect of LP-HFY11 through clinical practice, in order to fully study the mechanism of LP-HFY11.

## Figures and Tables

**Table 1:** Primer sequence for the reverse transcription-polymerase chain reaction in this study.

Gene	Primer Sequence
*CFTR*	F: 5′-CCCTTCGGCGATGCTTTTTC-3′
	R: 5′-AAGCCTATGCCAAGGTAAATGG-3′
*EGFR*	F: 5′-GCCATCTGGGCCAAAGATACC-3′
	R: 5′-GTCTTCGCATGAATAGGCCAAT-3′
*TGFβ1*	F: 5′-CCACCTGCAAGACCATCGAC-3′
	R: 5′-CTGGCGAGCCTTAGTTTGGAC-3′
*NHE1*	F: 5′-GACGAAGGTCCAGTTCCAGGC-3′
	R: 5′-GCCAACATCTCCCACAAGTCC-3′
*NHE4*	F: 5′-CTGGGTGAAACGGGTGATAAA-3′
	R: 5′-GGACATTTTGGCTGGATGTG-3′
*GAPDH*	F: 5′-AATGGATTTGGACGCATTGGT-3′
	R: 5′-TTTGCACTGGTACGTGTTGAT-3′

**Table 2:** In vitro resistance of *Lactiplantibacillus plantarun* HFY11.

Strain	Viability in pH 3.0 artificial gastric juice (%) NO (μmol/gprot)	Growth efficiency in 0.3% bile salt (%) MDA (nmol/mg) (nmol/mg)
LP-HFY11	82.15% ± 1.87	51.35 ± 1.08

## Data Availability

The data presented in this study are available on request from the corresponding author.

## References

[1] Dowarah R., Verma A.K., Agarwal N., Patel B.H.M., Singh P. (2017). Effect of swine based probiotic on performance, diarrhoea scores, intestinal microbiota and gut health of grower-finisher crossbred pigs. Livest. Sci..

[2] Vilkman K., Lääveri T., Pakkanen S.H., Kantele A. (2018). Stand-by antibiotics encourage unwarranted use of antibiotics for travelers’ diarrhea: A prospective study. Travel Med. Infect. Dis..

[3] Kmietowicz Z. (2013). Probiotics do not prevent diarrhoea caused by antibiotics in older people, study finds. BMJ.

[4] Li Z., Jia D., Liu J., Dragon K., Jin M., Wang J., Liu A., Wang J., Liu Z., Guan G. (2019). Antibiotic susceptibility and antimicrobial test of newly isolated Lactobacillus plantarum in vitro. Chin. Veter. Sci..

[5] Chen M., Wen C., Zhang J., Tang J., Wang L., Ding W. (2017). Probiotic properties in vitro and in vivo of antioxidative lactic acid bacteria from yak yogurt in Tibetan Plateau. Food Sci..

[6] Liang X., Tang C., Lei J., Shen F., Chen D. (2022). Screening and identification of bacteriocin-producing lactic acid bacteria from traditional yak yogurt. China Brew..

[7] Rishi P., Bharrhan S., Singh G., Kaur I.P. (2011). Effect of Lactobacillus plantarum and L-arginine against endotoxin-induced liver injury in a rat model. Life Sci..

[8] Hu T., Chen R., Qian Y., Ye K., Long X., Park K.Y., Zhao X. (2022). Antioxidant effect of Lactobacillus fermentum HFY02-fermented soy milk on D-galactose-induced aging mouse model. Food Sci. Hum. Well..

[9] Lukic J., Strahinic I., Milenkovic M., Golic N., Kojic M., Topisirovic L., Begovic J. (2013). Interaction of Lactobacillus fermentum BGHI14 with rat colonic mucosa: Implications for colitis induction. Appl. Environ. Microbiol..

[10] Groschwitz K.R., Hogan S.P. (2009). Intestinal barrier function: Molecular regulation and disease pathogenesis. J. Allergy Clin. Immunol..

[11] Brynskov J., Foegh P., Pedersen G., Ellervik C., Kirkegaard T., Bingham A., Saermark T. (2002). Tumour necrosis factor alpha converting enzyme (TACE) activity in the colonic mucosa of patients with inflammatory bowel disease. Gut.

[12] Johnston B.C., Supina A., Ospina M., Vohra S. (2008). Cochrane review: Probiotics for the prevention of pediatric antibiotic-associated diarrhea. Evid. Based Child Health A Cochrane Rev. J..

[13] Li J., Zhen H., Li Y., Wang N., Yang S., Jiang Z. (2023). Regulatory effect and mechanism of konjac mannan oligosaccharides on atherosclerosis in ApoE-/-mice. J. Food Sci. Technol..

[14] Wang M.W., She F., Song J., Liu Y.Q., Long X.Y., Zhao X., Hong H.Q. (2023). Preventive effect of *Lactiplantibacillus plantarun* YS1 isolated from naturally fermented yoghurt on carrageenan-induced thrombosis in mice. Acta Aliment..

[15] Patel S., Ganbold K., Cho C.H., Siddiqui J., Yildiz R., Sparman N., Sadeh S., Nguyen C.M., Wang J., Whitelegge J.P. (2024). Transcription factor PATZ1 promotes adipogenesis by controlling promoter regulatory loci of adipogenic factors. Nat. Communicat..

[16] Goubet A.G., Rouanne M., Derosa L., Kroemer G., Zitvogel L. (2023). From mucosal infection to successful cancer immunotherapy. Nat. Rev. Urol..

[17] Garey K.W., Salazar M., Shah D., Rodrigue R., DuPont H.L. (2008). Rifamycin antibiotics for treatment of Clostridium difficile-associated diarrhea. Ann. Pharm..

[18] Zhang S.R., Li X.D., Tian Q. (2016). Clinical effect of bifidobacterium tetravaccine tablets in the infantile non-infectious diarrhea and its impact in the expression of serum IL-6 and IL-17. China Med. Pharm..

[19] Zhou Q., Wu Z.Q., Li R.Z., Liu J., Wang C.K. (2008). The reparation effect of dried *Periplaneta americana* L. meal on mice intestinal dysfunction from diarrhea in a model study. Fujian J. Agri. Sci..

[20] Tang H.M., Fang C.F., Liao X.H., Li D.T., Yao N., He J.L. (2008). Expression of NPY and 5-HT in rat model of diarrhea-predominant irritable bowel syndrome. Chin. Pharmacol. Bull..

[21] Shimokawa C., Kabir M., Taniuchi M., Mondal D., Kobayashi S., Ali I.K., Sobuz S.U., Senba M., Houpt E., Haque R. (2012). Entamoeba moshkovskii is associated with diarrhea in infants and causes diarrhea and colitis in mice. J. Infect. Dis..

[22] Pang T., Su X., Wakabayashi S., Shigekawa M. (2001). Calcineurin homologous protein as an essential cofactor for Na^+^/H^+^ exchangers. J. Biol. Chem..

[23] Cabral J.M., Grácio D., Soares-da-Silva P., Magro F. (2015). Short- and long-term regulation of intestinal Na^+^/H^+^ exchange by Toll-like receptors TLR4 and TLR5. Am. J. Physiol. Gastrointest. Liver Physiol..

[24] Troncone E., Marafini I., Stolfi C., Monteleone G. (2018). Transforming growth factor-β1/Smad7 in intestinal immunity, inflammation, and cancer. Front. Immunol..

[25] Dixméras I., Lapouméroulie C., Tallec L.P., Bens M., Elion J., Vandewalle A., Denamur E. (1998). CFTR regions containing duodenum specific DNase I hypersensitive sites drive expression in intestinal crypt cells but not in fibroblasts. Biochem. Biophy. Res. Communicat..

[26] Mogna L., Del Piano M., Deidda F., Nicola S., Soattini L., Debiaggi R., Sforza F., Strozzi G., Mogna G. (2012). Assessment of the in vitro inhibitory activity of specific probiotic bacteria against different Escherichia coli strains. J. Clin. Gastroenterol..

[27] Borthakur A., Bhattacharyya S., Kumar A., Anbazhagan A.N., Tobacman J.K., Dudeja P.K. (2013). Lactobacillus acidophilus alleviates platelet-activating factor-induced inflammatory responses in human intestinal epithelial cells. PLoS ONE.

[28] Belizário J.E., Napolitano M. (2015). Human microbiomes and their roles in dysbiosis, common diseases, and novel therapeutic approaches. Front. Microbiol..

[29] Doron S.I., Hibberd P.L., Gorbach S.L. (2008). Probiotics for prevention of antibiotic-associated diarrhea. J. Clin. Gastroenterol..

[30] Teitelbaum J.E. (2005). Probiotics and the treatment of infectious diarrhea. Pediatr. Infect. Dis. J..

[31] Marteau P., Seksik P. (2004). Tolerance of probiotics and prebiotics. J. Clin. Gastroenterol..

[32] Allen S.J., Martinez E.G., Gregorio G.V., Dans K.F. (2010). Probiotics for treating acute infectious diarrhoea. Cochrane Database Syst. Rev..

[33] Cremonini F., Di Caro S., Santarelli L., Gabrielli M., Candelli M., Nista E.C., Lupascu A., Gasbarrini G., Gasbarrini A. (2002). Probiotics in antibiotic-associated diarrhoea. Dig. Liver Dis..

[34] Macfarlane. S., Macfarlane G.T., Cummings J.H. (2006). Review article: Prebiotics in the gastrointestinal tract. Aliment. Pharmacol. Ther..

